# Gender and Clinical Status in Burnout in Medicine

**DOI:** 10.1001/jamanetworkopen.2024.6575

**Published:** 2024-04-11

**Authors:** Deesha Sarma, David T. Chiu, Alexa B. Kimball

**Affiliations:** 1 Department of Emergency Medicine, Beth Israel Deaconess Medical Center, Boston, Massachusetts; 2 Department of Dermatology, Beth Israel Deaconess Medical Center, Boston, Massachusetts

## Abstract

This survey study assesses feelings of satisfaction, stress, and burnout by gender and clinical status among health care workers at a single academic center.

## Introduction

There has been increasing focus on physician well-being within the past decade.^1^ A 2023 survey found burnout was driving physicians to cut work hours, with 40% of those surveyed reporting they had or were planning to reduce clinical hours.^2^ Transition to part-time clinical status has been proposed as a potential solution. The American Medical Association (AMA) conducts yearly well-being assessments at health care institutions across the country called the *Organizational Biopsy*. Institution-specific 2023 results indicated part-time employees had lower rates of stress and burnout and higher satisfaction. While it has been established that women physicians have higher rates of burnout than their men counterparts, little research has delved into whether part-time status can have protective associations against burnout among women physicians.^3,4^ Therefore, the goal of this study was to evaluate key indicators in the AMA Organizational Biopsy by gender and clinical status.

## Methods

For this survey study, data were provided by the AMA from the institution-specific results of the 2023 Organizational Biopsy. The report adheres to best practice guidelines by publishing information such as survey mode, full question text, and answer choices through the AMA. The study site was an urban, academic teaching institution located in the Northeast, and the hospital institutional review board determined that the study qualified as exempt from review and informed consent because the proposed activity did not constitute human participants research. The survey was distributed via email to all employees with a response window from May 12 through June 5, 2023. All responses were deidentified and aggregated anonymously. Participants self-identified gender (man, woman, trans female, trans male, or nonbinary), age, ethnicity, department, and clinical status (full or part-time). Markers of well-being were organized into 5 key performance indicators (KPIs): satisfaction, stress, burnout, likelihood of leaving in 2 years, and feeling valued by the organization. We used χ^2^ and logistic regression to analyze the results using SAS software version 9.4 (SAS Institute). *P* values were 2-sided, and statistical significance was set at *P* = .05. Data were analyzed from November 2023 through January 2024.

## Results

A total of 742 employees responded, a 40% response rate. More than two-thirds of respondents were full-time employees (572 employees [77%]), with 170 employees (23%) working part-time. Part-time employees reported working a mean of 50.4 hours per week, and full-time employees worked a mean of 62.2 hours per week. Table 1 demonstrates KPIs by clinical status.

**Table 1.** Key Performance Indicators Measurements by Full-Time vs Part-Time Clinical Status

Key performance indicator	Respondents, No. (%) [95% CI]		*P* value
	Full-time (n = 572)	Part-time (n = 170)	
Satisfaction (agree strongly, agree)	387 (68) [63-72]	125 (74) [66-81]	.36
Stress (agree strongly, agree)	290 (51) [45-57]	69 (41) [29-53]	.06
Burnout (experiencing ≥1 symptom of burnout)	283 (50) [44-55]	69 (41) [29-53]	.10
Likelihood to leave in 2 y (moderately, likely, definitely)	241 (43) [37-49]	75 (45) [34-56]	.72
Feeling valued by organization (to a great extent, moderately)	284 (50) [44-56]	97 (57) [47-67]	.17

The differences in KPIs between men and women were not statistically significant. When analyzing KPIs by clinical status and gender, part-time women had significantly lower satisfaction and feelings of being valued and higher stress. Among full-time workers, women had statistically significantly lower satisfaction and higher levels of burnout, stress, and likelihood of leaving. Table 2 demonstrates KPIs by gender for both full-time and part-time employees.

**Table 2.** Key Performance Indicators Measurements by Men vs Women

Key performance indicator	Full-time			Part-time		
	Respondents, No. (%) [95% CI]		*P* value	Respondents, No. (%) [95% CI]		*P* value
	Women (n = 251)	Men (n = 299)		Women (n = 102)	Men (n = 65)	
Satisfaction (agree strongly, agree)	180 (71.7) [65.1-78.3]	203 (67.9) [61.5-74.3]	.003	67 (65.7) [54.3-77.1]	55 (84.6) [75.1-94.1]	.02
Stress (agree strongly, agree)	138 (55.0) [46.7-63.3]	137 (45.8) [37.5-54.1]	.03	47 (46.1) [31.8-60.4]	21 (32.4) [12.4-52.4]	.01
Burnout (experiencing ≥1 symptom of burnout)	132 (52.6) [44.1-61.1]	133 (44.4) [36.0-52.8]	.04	45 (44.1) [29.6-58.6]	24 (36.9) [17.6-56.2]	.13
Likelihood to leave in 2 y (moderately, likely, definitely)	106 (42.5) [33.1-51.9]	118 (40.4) [31.5-49.3]	.03	46 (46.0) [31.6-60.4]	29 (46.0) [27.9-64.1]	.73
Feeling valued by organization (to a great extent, moderately)	126 (50.2) [41.5-58.9]	151 (50.5) [42.5-58.5]	.12	49 (48.1) [34.1-62.1]	46 (70.7) [57.5-83.9]	.03

## Discussion

This initial institution-specific survey study found significant differences in work satisfaction and burnout between male and female employees, even when accounting for full-time and part-time clinical status. One inference is women who transition to part-time make trade-offs to balance family commitments, whereas men pursue other career-related interests or reduce their workload. The likelihood of leaving was the same between groups, which could point to women’s resiliency and commitment to medicine.

Part-time employees worked fewer clinical hours, approximately 1 less day per work week, but spent similar hours on nonclinical work, like administrative tasks, which have been shown to drive discontent.^5^ In the end, the number of hours, not clinical status, may be critical to satisfaction, as research has found half of physicians who reported burnout worked more than 51 hours per week.^6^

The study has several limitations, including a lower response rate, which introduces nonresponse bias. In addition, as a single-center study, the results may not be generalizable to other institutions. Overall, the data suggest that part-time status was not necessarily a protective strategy for women in particular. Increased research is warranted to clarify the stressors of part-time women to support them in the work environment.

## Notes

Supplement.
Data Sharing Statement
