# Supplementary material for: Gender and Clinical Status in Burnout in Medicine
Source: JAMA Netw Open. 2024 Apr 11;7(4):e246575. doi: 10.1001/jamanetworkopen.2024.6575 (PMC11009818; doi:10.1001/jamanetworkopen.2024.6575)
Supplement: Supplement. — Data Sharing Statement [file jamanetwopen-e246575-s001.pdf]

Data Sharing Statement

Sarma. Gender and Clinical Status in Burnout in Medicine. *JAMA Netw Open*. Published April 11, 2024. doi:10.1001/jamanetworkopen.2024.6575

Data
Data available: No

Additional Information
Explanation for why data not available: Data is proprietary to the AMA and was collected for its annual Organizational Biopsy, so they would need to agree before any data is made available or shared
